# A 31-bp indel in the 5′ UTR region of *GNB1L* is significantly associated with chicken body weight and carcass traits

**DOI:** 10.1186/s12863-020-00900-z

**Published:** 2020-08-26

**Authors:** Tuanhui Ren, Ying Yang, Wujian Lin, Wangyu Li, Mingjian Xian, Rong Fu, Zihao Zhang, Guodong Mo, Wen Luo, Xiquan Zhang

**Affiliations:** 1 Department of Animal Genetics, Breeding and Reproduction, College of Animal Science, South China Agricultural University, Guangzhou, 510642 Guangdong China; 2 Guangdong Provincial Key Lab of Agro-Animal Genomics and Molecular Breeding, and Key Laboratory of Chicken Genetics, Breeding and Reproduction, Ministry of Agriculture, Guangzhou, 510642 Guangdong China; 3 College of Life Science, Foshan University, Foshan, 528231 Guangdong China

**Keywords:** Chicken, *GNB1L*, Indel, Growth traits, Carcass traits

## Abstract

**Background:**

G-protein subunit beta 1 like (*GNB1L*) encodes a G-protein beta-subunit-like polypeptide. Chicken *GNB1L* is upregulated in the breast muscle of high feed efficiency chickens, and its expression is 1.52-fold that in low feed efficiency chickens. However, no report has described the effects of *GNB1L* indels on the chicken carcass and growth traits.

**Results:**

This study identified a 31-bp indel in the 5′ untranslated region (UTR) of *GNB1L* and elucidated the effect of this gene mutation on the carcass and growth traits in chickens. The 31-bp indel showed a highly significant association with the body weight at 8 different stages and was significantly correlated with daily gains at 0 to 4 weeks and 4 to 8 weeks. Similarly, the mutation was significantly associated with small intestine length, breast width, breast depth and breast muscle weight. Moreover, *DD* and *ID* were superior genotypes for chicken growth and carcass traits.

**Conclusions:**

These results show that the 31-bp indel of *GNB1L* significantly affects chicken body weight and carcass traits and can serve as a candidate molecular marker for chicken genetics and breeding programs.

## Background

Compared with pigs and cattle, chickens have high feed efficiency and a short growth period, and chicken meat is the second-largest meat product after pork in China [1]. Therefore, chicken breeds play an indispensable role in husbandry. The body weight of animals as an economic trait can directly reflect the balance of nutrients through digestive absorption and energy metabolism, leading to skeletal growth and lean or fat deposition [2, 3].

G-protein subunit beta 1 like (*GNB1L*) encodes a G-protein beta-subunit-like polypeptide that lacks homology with known proteins [4]. In humans, the hemizygous deletion of *GNB1L* can cause sensorimotor gating defects, which are related to schizophrenia and other serious mental diseases [5, 6]. Changes in *GNB1L* expression are also associated with markers related to psychosis [7]. In the study of chickens, the candidate gene *GNB1L* for the ear-tufted trait was verified by GWAS and haplotype analysis [8]. Additionally, *GNB1L* was shown to be related to higher feed efficiency, it is upregulated in the breast muscle of high feed efficiency chickens, and its expression is 1.52-fold that of low feed efficiency chickens [9]. However, no report has described the effects of *GNB1L* indels on chicken growth and carcass traits.

Gene variants, such as single-nucleotide polymorphisms (SNPs) and insertion-deletions (indels), are widely distributed in an animal’s genome, as reported in many studies on humans and livestock animals [10, 11]. Compared with SNPs, the genotyping of large-fragment indels has higher efficiency [12]. Indel mutations also play important roles in many aspects of animal economic traits. A 10-bp indel exists in the *PAX7* promoter region, which is located at the binding site of *ZNF219*, and the homozygous deletion genotype upregulated the expression and promoter activity of *PAX7*, which, in turn, affected the early growth traits of cattle [13]. A 19-bp indel mutation in the *PLAGI* intron region affected the growth traits of Chinese cattle [14]. The 16-bp indel in the 5′ untranslated regions (UTR) of *ZNF132* significantly affected the body length of the Hainan black goat [3]. Recent studies have revealed that the 11-bp indel in the *DNMT3B* intron region was significantly correlated with the litter size at first parity in the goat [15]; two indels (P2–16 bp and P14–15 bp) of *DSCAML1* were significantly related to sperm quality in the male goat, and three indels of *DSCAML*1 were significantly correlated with the litter size at first parity in female goat [16]. A study has shown that the 13-bp indel mutation in the 3′ UTR of *DGAT2* affected its expression and fat deposition in porcine [17]. In poultry research, two novel indels in *QPCTL* significantly affected chicken carcass traits and body weight at 5 different weeks of age [18]; the indel of *CDKN3* was significantly associated with chicken carcass and growth traits [19]; a 22-bp indel of *ZNF764L* was significantly related to chicken birth weight, body slanting length, chest breadth and subcutaneous fat weight [20]. A 65-bp indel in the chicken *GOLGB1* intron significantly affected body weight and carcass traits at 13 weeks [21]. The 80-bp indel in *PRLR* was significantly associated with chicken leg weight, body weight and shank length [22].

In the present study, we confirmed a 31-bp indel in *GNB1L* from 10× whole-genome resequencing data of ten XH and ten RW chickens (data unpublished) (EVA accession number: PRJEB36864). Chicken *GNB1L* is located on chromosome 15 and comprises 15 exons. Furthermore, 80 indels were found in *GNB1L* in the Ensembl database (http://asia.ensembl.org/Gallus_gallus/Gene/Variation_Gene/Table?db=core;g=ENSGALG00000001925;r=15:1232691-1273276;t=ENSGALT00000002979). However, there are no report and verification about the Indel of chicken *GNB1L*. This study was aimed to verify the indel mutation of *GNB1L*, clarify the effect of the *GNB1L* indel on chicken economic traits, and analyze *GNB1L* expression in different tissues and in leg and breast muscle tissues at different embryonic development stages. Additionally, we examined the distribution of the 31-bp indel in different populations. These results indicated that the 31-bp indel mutation in *GNB1L* could be used as a candidate molecular marker for chicken growth traits and provided a reference for the molecular breeding of chickens.

## Results

### Polymorphism detection and genotyping

A novel 31-bp indel polymorphism in the 5′ UTR region of *GNB1L* was observed by DNA sequencing (Figure S1) (TSINGKE, Guangzhou, China). All PCR amplification products were detected using 3.0% agarose gel electrophoresis, which revealed three genotypes, the 301-bp homozygous *DD* genotype, the heterozygous *ID* genotype (332 bp and 301 bp) and the 332-bp homozygous *II* genotype (Figure S2).

### Genetic diversity of the 31-bp indel in different populations

The genetic parameters, allele frequencies and genotype frequencies of seven different breeds and the F2 population were analyzed (Table 1). The results suggest that the *D* allele frequency is lower than that of *I* in all breeds, except in LS chicken. Additionally, we counted different genotype distributions among the dual-purpose chickens (ND, GX, WC, QY and LS), F2 population, commercial broilers (RW) and commercial layers (ISA). The percentage of the *DD* genotype was the lowest in all breeds (Figure S3). The results of χ^2^ test suggest that the genotype frequencies of F2, ND and RW are not in HWE (*P* < 0.05), and WC, QY, ISA and LS were in HWE (*P* > 0.05). The values of He were from 0.46 to 0.50, and those of Ne were from 1.85 to 1.99. The smallest and largest values of PIC were 0.35 and 0.37, respectively. The results revealed that the 31-bp indel of *GNB1L* represents intermediate polymorphism and the lack of high genetic diversity among all the populations (Table 1).

**Table 1:** Genotypic and allelic frequencies and genetic parameters of chicken *GNB1L*

Breeds/n	Genotypic and allelic frequencies					He	Ne	PIC	*P*-value
	*DD*	*ID*	*II*	*D*	*I*				
F2/360	0.20	0.35	0.45	0.375	0.625	0.47	1.89	0.36	0.00
ND/95	0.08	0.61	0.31	0.385	0.615	0.48	1.91	0.36	0.01
RW/55	0.29	0.35	0.36	0.465	0.535	0.50	1.99	0.37	0.02
ISA/64	0.16	0.53	0.31	0.425	0.575	0.49	1.95	0.37	0.48
GX/71	0.13	0.46	0.41	0.36	0.64	0.46	1.85	0.35	0.93
WC/65	0.2	0.49	0.31	0.445	0.555	0.49	1.97	0.37	0.98
QY/70	0.15	0.45	0.4	0.375	0.625	0.47	1.88	0.36	0.76
LS/39	0.30	0.61	0.08	0.605	0.395	0.47	1.90	0.36	0.06

*F2* F2 resource population (F2; *n* = 360), *ND* Ningdu chickens, *RW* Recessive White Rock chickens, *ISA* ISA Brown laying hen, *GX* Guangxi chickens, *WC* Wenchang chickens, *QY* Qingyuan Partridge chickens, *LS* Lushi chickens, *Ne* effective allele numbers, *He* gene heterozygosity, *PIC* polymorphism information content, *P*-value *P*-value of Hardy–Weinberg equilibrium

### Genetic differentiation of the 31-bp indel

The results of differential selection suggest medium genetic differentiation between LS and QY and between LS and GX (0.05 < Fst < 0.15). Moreover, we observed little genetic differentiation among the other breeds (Fst < 0.05; Table S1).

### Correlation between the *GNB1L* 31-bp indel and economic traits

The mixed model was used to analyze the correlation between the genotypes and economic traits. Three genotypes showed a significant correlation with 11 chicken growth traits, and a highly significant association with 9 growth traits (Table 2). In particular, different genotypes showed a highly significant correlation with body weight at 7, 14, 21, 28, 35, 42, 49 and 56 weeks and daily gains at 0 to 4 weeks (*P* < 0.01) and were significantly related to daily gains at 4 to 8 weeks and shank length at 49 weeks (*P* < 0.05) (Table 2). Importantly, the *DD* and *ID* genotypes were greater than the *II* genotype in all the related growth traits.

**Table 2:** Association analysis of the *GNB1L* 31-bp indel with growth traits in the Xinghua × Recessive White Rock F2 populations

Traits	Mean ± SE			*P*-value
	DD	ID	II	
BW0 (g)	30.2 ± 0.3	29.9 ± 0.2	29.7 ± 0.2	0.359
BW7 (g)	61.9 ± 1.1^a^	60.6 ± 0.8^a^	58.1 ± 0.7^b^	0.004
BW14 (g)	130.0 ± 2.1^a^	128.2 ± 1.6^a^	119.7 ± 1.4^b^	0.000
BW21 (g)	221.2 ± 3.9^a^	219.5 ± 2.9^a^	203.2 ± 2.6^b^	0.000
BW28 (g)	326.3 ± 6.2^a^	319.6 ± 4.6^a^	300 ± 4.1^b^	0.000
BW35 (g)	459.1 ± 9.0^a^	449.8 ± 6.8^a^	423.2 ± 6.1^b^	0.001
BW42 (g)	599.0 ± 12.5^a^	594.5 ± 9.2^a^	552.3 ± 8.2^b^	0.000
BW49 (g)	739.4 ± 14.4^a^	735.3 ± 10.7^a^	682.5 ± 9.6^b^	0.000
BW56 (g)	885.0 ± 17.0^ab^	889.9 ± 12.6^a^	837.9 ± 11.2^b^	0.004
BW63 (g)	1051.1 ± 23.0	1032.6 ± 19.2	993.2 ± 16.5	0.088
BW70 (g)	1117.6 ± 26.1	1161.8 ± 17.9	1120.7 ± 15.9	0.180
BW77 (g)	1327.9 ± 29.6	1359.6 ± 20.2	1321.2 ± 18.1	0.353
BW84 (g)	1475 ± 38.2	1514.8 ± 27.7	1487.4 ± 22.7	0.640
SL42 (mm)	61.4 ± 0.6	61.1 ± 0.4	60.1 ± 0.4	0.063
SL49 (mm)	69.4 ± 0.7	67.4 ± 0.7	67.1 ± 0.5	0.033
SL56 (mm)	72.9 ± 0.6	72.8 ± 0.4	71.9 ± 0.4	0.205
SL63 (mm)	79.9 ± 1.4	78.2 ± 1.1	79.3 ± 0.9	0.608
SL70 (mm)	82.5 ± 0.8	83.2 ± 0.5	81.7 ± 0.5	0.111
SL77 (mm)	89.4 ± 1.3	88.4 ± 1.0	88.5 ± 0.8	0.827
SL84 (mm)	88.7 ± 0.9	89.6 ± 0.7	88.9 ± 0.6	0.651
SD42 (mm)	7.9 ± 0.1	8.0 ± 0.1	7.8 ± 0.1	0.174
SD49 (mm)	8.6 ± 0.1	8.6 ± 0.1	8.4 ± 0.1	0.472
SD56 (mm)	8.8 ± 0.1	8.9 ± 0.1	8.7 ± 0.1	0.258
SD63 (mm)	9.3 ± 0.2	9.3 ± 0.2	9.3 ± 0.1	0.892
SD70 (mm)	9.4 ± 0.1	9.5 ± 0.1	9.4 ± 0.1	0.670
SD77 (mm)	9.7 ± 0.2	10.0 ± 0.2	10.0 ± 0.1	0.404
SD84 (mm)	10.0 ± 0.2	10.1 ± 0.1	10.0 ± 0.1	0.725
0–4 DG (g/week)	10.6 ± 0.2^a^	10.3 ± 0.2^a^	9.6 ± 0.1^b^	0.000
4–8 DG (g/week)	20.1 ± 0.5	20.3 ± 0.3	19.2 ± 0.3	0.043

Note: SE = standard error of the mean; BW0, 7, 14, 21, 28, 35, 42, 49, 56, 63, 70, 77 and 84 = body weights at the ages of 0, 7, 14, 21, 28, 35, 42, 49, 56, 63,70, 77 and 84 days, respectively; SL42, 49, 56, 63, 70, 77 and 84 = shank lengths at the ages of 42, 49, 56, 63, 70, 77 and 84 days, respectively; SD42, 49, 56, 63, 70, 77 and 84 = shank diameters at the ages of 42, 49, 56, 63,70, 77 and 84 days, respectively; 0 to 4 and 4 to 8 DG = daily gains at 0 to 4 and 4 to 8 weeks, respectively. Means with different superscripts indicate highly significant differences (different lowercase letters indicate *P* < 0.01; the same letters indicate *P* > 0.01)

Notably, the 31-bp indel displayed a highly significant correlation with breast width, breast depth, breast muscle weight and small intestine length in carcass traits, and was significantly correlated with fat cingula width (Table 3). Interestingly, the *DD* and *ID* genotypes were greater than the *II* genotype in all the related carcass traits. In the association analysis of the 31-bp indel and meat quality traits, the different genotypes showed a significant correlation with the dry matter content of leg muscle and a critical correlation with the crude fat content of leg muscle (Table S2).

**Table 3:** Association analysis of the *GNB1L* 31-bp indel with carcass traits in the Xinghua × Recessive White Rock F2 populations

Traits	Mean ± SE			*P*-value
	*DD*	*ID*	*II*	
LWS (kg)	1.5 ± 0.0	1.5 ± 0.0	1.5 ± 0.0	0.522
BWH (mm)	67.3 ± 0.68^ab^	67.9 ± 0.5^a^	65.8 ± 0.5^b^	0.006
BP (mm)	96.8 ± 1.023^ab^	96.7 ± 0.8^a^	93.9 ± 0.7^b^	0.009
BSL (cm)	22.9 ± 0.2	23.1 ± 0.1	22.9 ± 0.1	0.606
BAW (°)	60.9 ± 0.6	60.8 ± 0.4	60.3 ± 0.4	0.599
CW (g)	1353.2 ± 27.1	1379.6 ± 20.2	1347.3 ± 18.0	0.477
SFT (mm)	4.1 ± 0.1	4.2 ± 0.1	4.1 ± 0.1	0.837
FCW (mm)	11.2 ± 0.4	11.8 ± 0.3	12.5 ± 0.3	0.038
SEW (g)	1241.3 ± 24.3	1264.0 ± 18.2	1228.7 ± 16.2	0.347
EW (g)	1073.8 ± 21.6	1097.0 ± 16.1	1066.4 ± 14.4	0.358
BMW (g)	95.9 ± 2.05^a^	95.5 ± 1.5^a^	88.5 ± 1.4^b^	0.001
LMW (g)	115.0 ± 2.6	120.1 ± 1.9	117.3 ± 1.7	0.268
WB (g)	66.2 ± 1.3	67.0 ± 1.0	64.7 ± 0.9	0.193
AFW (g)	29.7 ± 2.2	27.6 ± 1.6	27.4 ± 1.4	0.651
SIL (cm)	144.9 ± 1.952^a^	140.3 ± 1.5^ab^	136.7 ± 1.31^b^	0.002

*SE* standard error of the mean, *LWS* live weight before slaughter, *BWH* breast width, *BP* breast depth, *BSL* body slanting length, *BAW* breast angle width, *CW* carcass weight, *SFT* subcutaneous fat thickness, *FCW* fat cingula width, *EW* eviscerated weight, *SEW* semi-eviscerated weight, *BMW* breast meat weight, *LMW* leg meat weight, *AFW* abdominal fat weight, *WW* wing weight, *SIL* small intestine length

Means with different superscripts indicate highly significant differences (different lowercase letters indicate *P* < 0.01; and the same letters indicate *P* > 0.01)

### *GNB1L* expression in chickens

**Fig. 1:**
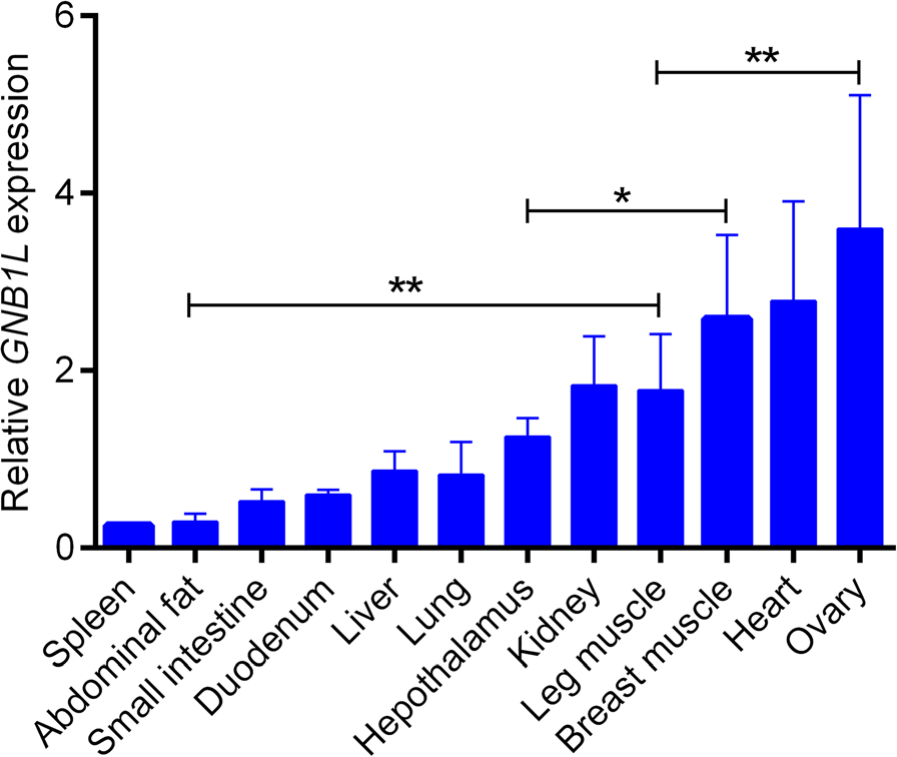
Relative expression patterns of *GNB1L* in different tissues. The data are expressed as means ± SD (*n* = 3)

**Fig. 2:**
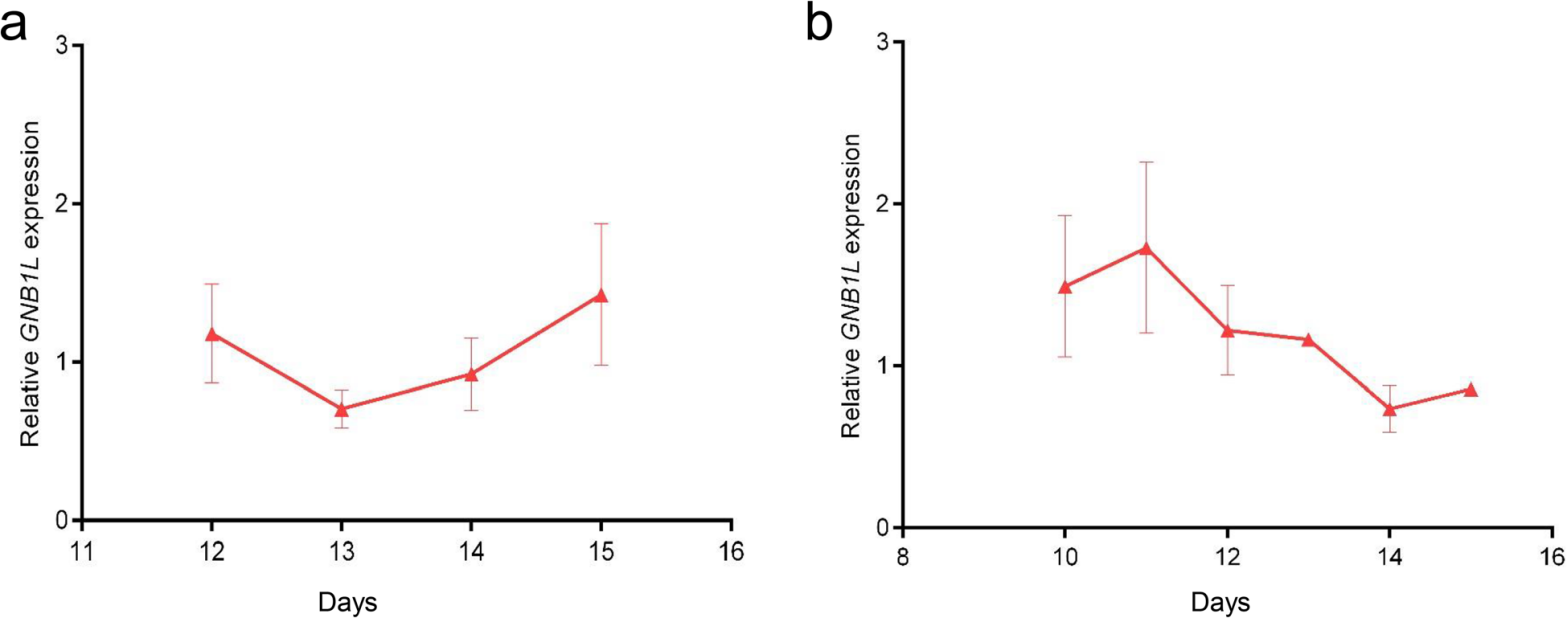
**a.** Expression of *GNB1L* in leg muscle at different embryonic stages. **b.** Expression of *GNB1L* in breast muscle at different embryonic stages. The data are expressed as means ± SD (*n* = 3)

*GNB1L* expression in 12 tissues of 20-week-old QY spotted-brown chickens was detected by qPCR. *GNB1L* was found to be relatively highly abundant in the heart, breast muscle, leg muscle, kidney and ovary and showed low expression levels in the small intestine, spleen, liver, lung, and abdominal fat (Fig. 1). Furthermore, *GNB1L* expression increased first and then decreased in breast muscle at different embryonic stages, and the expression level decreased first and then increased in leg muscle at different embryonic stages (Fig. 2a, b).

### Transcription factor prediction in the *GNB1L* 31-bp indel

The transcription binding sites in the 31-bp indel of *GNB1L* were analyzed using an online prediction website, and the results revealed five potential transcription factors (NF-1, SP1, T3R, RAR-α and GR) (Figure S4).

## Discussion

The allelic frequency of genes can reflect the genetic diversity between different groups, indicating that new mutations are introduced to some extent [2, 23]. In recent decades, the breeding of commercial broilers and layers has focused on growth and reproductive traits, respectively. In these commercial breeds, dominant genotypes for specific traits may be selected for breeding. Moreover, manual selection also determines the distribution and amount of genetic variation during domestication [23]. In this study, the *I* allele was the predominant allele in the ND, GX, WC, QY, F2, RW and ISA chickens, but not in LS chickens. The results showed that LS chickens might undergo different selection pressure during evolutionary processes compared with other chickens. Interestingly, the LS chicken is the only breed that can produce blue eggs among these breeds [24].

The body weight of chickens is a heritable trait with approximately 0.24–0.47% heritability during growth [25]. Compared with commercial broilers, Chinese domestic broilers have a relatively low growth rate and body weight. Therefore, we studied the correlation between the 31-bp indel in the *GNB1L* 5′ UTR region and F2 population carcass and growth traits. The 31-bp indel showed a highly significant correlation with the body weight at 8 different stages (Table 2). Moreover, the three different genotypes were significantly correlated with daily gains at 0 to 4 weeks and 4 to 8 weeks, and shank length at 49 weeks (Table 2). The *DD* and *ID* genotypes were greater than the *II* genotype in all the related growth traits, with the *DD* genotype showing the highest weights at 7, 14, 21, 28, 35, 42 and 49 weeks, but not at 56 weeks. Interestingly, *DD* is the dominant genotype for daily gains at 0 to 4 weeks and 4 to 8 weeks. We hypothesized that the *DD* and *ID* genotypes might have a higher feed conversion ratio during chicken development. In summary, the *II* genotype is a disadvantaged genotype in all growth traits.

Chinese domestic chickens have a good carcass yield, with breast muscles accounting for approximately 30% of the carcass weight and the weight of muscles accounting for approximately 40% of the weight of the carcass [26]. Therefore, individuals with a larger breast width, breast depth and breast weight are also the breeding direction of local yellow-feathered broilers. The mutation was significantly related to breast width, breast muscle weight, breast depth and small intestine length of carcass traits (Table 3). Similarly, the *DD* and *ID* genotypes were greater than the *II* genotype in all the related carcass traits. Growing evidence suggests that the small intestine is mainly responsible for the efficient absorption and metabolic processing of nutrients, and the small intestinal villi are the main sites for absorbing nutrients [27]. Perhaps the longer length of the small intestine is helpful in improving the efficiency of animal absorption of food. We speculate that *GNB1L* 31-bp indel may affect the conversion efficiency of feed by affecting the length of the small intestine, ultimately leading to differences in carcass and growth traits of individuals with different genotypes. Previous research results also indicate that *GNB1L* is related to higher feed efficiency [9].

Studies have demonstrated that mutations in the 5′ UTR of some genes can affect gene expression [28, 29]. Furthermore, TFs are essential factors that regulate gene expression, and the prediction results of TFs showed five potential TFs (NF-1, SP1, T3R, RAR-α and GR) associated with the 31-bp *GNB1L* indel. We speculate that these TFs may be involved in the transcription of *GNB1L*, which leads to differences in the phenotype among the three genotypes. In this study, the expression of *GNB1L* was relatively highly abundant in the heart, leg muscle, breast muscle, kidney and ovary; other tissues had relatively low expression levels. Moreover, *GNB1L* expression increased first and then decreased in breast muscle at different embryonic stages, and decreased first and then increased in leg muscle. These results showed that *GNB1L* might be related to embryonic muscle development.

## Conclusion

For the first time, we found that *GNB1L*, a candidate gene for high feed efficiency, has a 31-bp indel in its 5′ UTR that is significantly related to chicken carcass and growth traits. Moreover, *DD* and *ID* are superior genotypes for the carcass and growth traits of chickens. Furthermore, we confirmed that *GNB1L* might be a candidate gene for a higher feed conversion rate. In summary, this study showed that *GNB1L* might be involved in chicken embryonic development and growth, and the 31-bp indel of *GNB1L* can serve as a candidate molecular marker for the genetics and breeding programs of chicken.

## Methods

### Animal samples and trait measurement

The DNA samples of 766 chickens were obtained from the following eight populations: Lushi chickens (LS, *n* = 39, 6 weeks), Ningdu chickens (ND, *n* = 95, 12 weeks), Wenchang chickens (WC, *n* = 65, 7 weeks), Qingyuan Partridge chickens (QY, *n* = 70, 7 weeks), Recessive White Rock chickens (RW, *n* = 55, 7 weeks), ISA Brown laying hen (ISA, *n* = 54, 20 weeks), Guangxi chickens (GX, *n* = 71, 12 weeks) and F2 population (F2, *n* = 360, 13 weeks). These DNA samples were all from the chicken breed resource library maintained in our laboratory. Among the eight different breeds, LS, ND, WC, QY and GX are domestic chicken breeds in China and RW and ISA are commercial broilers and layer hens, respectively. Additionally, the F2 resource population is a hybrid strain of RW and Xinghua (XH) chickens; XH chickens represent a slow-growing Chinese domestic chicken. In our laboratory, 2 mL of 5% pentobarbital was injected intraperitoneally into the chicken (No. 57–33-0; Chinese Academy of Sciences, Beijing Siyuan Technology Co., Ltd.). After 2–3 min, the chicken was sacrificed by bleeding through the carotid artery. Data records about economic traits, as well as detailed information on the measuring methods, were available for the F2 population, as previously described [30].

Twelve different tissues were obtained from four QY chickens. Additionally, the breast muscle of six embryonic periods (E10–15) and leg muscle of four embryonic periods (E12–15) were used to detect relative *GNB1L* expression.

### cDNA synthesis and qRT–PCR

RNA was extracted using TRIzol (Takara, Dalian, China). Next, the RNA was reverse transcribed using the cDNA reverse transcription kit (Takara, Dalian, China) and then was subjected to PCR. Relative gene expression was calculated using the 2^–ΔΔCt^ method, and significance was determined using ANOVA followed by Duncan’s test. All the reactions were performed using three biological and technical repetitions. The relative expression of *GNB1L* using different tissues and embryo ages were analyzed by qRT–PCR. The qRT–PCR primers used for *GNB1L* and the internal control *β-actin* are listed in Table S3.

### Indel detection and diversity analysis of different breeds

A 31-bp indel was identified in *GNB1L* from whole-genome resequencing data of ten XH and ten RW chickens (unpublished data). Genotyping of the *GNB1L* 31-bp indel was performed by PCR amplification and gel electrophoresis in eight diverse populations. Blood samples were used to extract DNA, and the final DNA concentration used for amplification was diluted to 50 ng/μL. The *GNB1L* PCR primers based on the genome are listed in Table S3. Each 15-μL PCR amplification volume contained 1 μL of DNA, 1.5 μL of primer, 7.5 μL 2 × Taq Master mix (TSINGKE, Beijing, China), and 5 μL of double-distilled water. The PCR parameters were as follows: 95 °C for 3 min, 35 cycles at 95 °C for 30 s, 60 °C for 30 s, 72 °C for 30 s, and a final extension at 72 °C for 10 min. The PCR products after amplification were separated by 3.0% gel electrophoresis.

The genotype and allele frequencies of the mutation were calculated directly in different breeds. Hardy–Weinberg equilibrium (HWE) was analyzed using the SHEsis website (http://analysis.biox.cn). Moreover, the allele numbers (Ne), genetic indices of heterozygosity (He), effective polymorphism information content (PIC) and population differentiation were analyzed using PopGene software (Version 1.3.1) [31, 32].

### Transcription factor prediction

The transcription factors (TFs) in the 31-bp indel mutation of the 5′ UTR region of *GNB1L* were predicted using AliBaba software (Version 2.1) [24].

### Statistics

Association analysis of the F2 population was performed using SPSS 22.0 software, and two different models were used in the analysis. All the growth traits used Model I (Yijkl = μ + Gi + Sj + Hk + fl + eijkl), and all the carcass traits used Model II (Yijkl = μ + Gi + Sj + Hk + fl + b (Wijkl - $\overline{\mathrm{W}}$) + eijkl); the carcass weight served as a concomitant variable of Model II. Yijkl represents the observed value, μ is the overall population mean, fl is the fixed effect of family, Gi is the fixed effect of genotype, Hk is the fixed effect of hatch, Sj is the fixed effect of sex, b is the regression coefficient for carcass weight, $\overline{\mathrm{W}}$ is average slaughter weight, Wijkl represents the individual slaughter weight, and eijkl represents the random error in the two models. Significance was set at a *P*-value < 0.05, and Bonferroni’s test was performed for multiple comparisons [18].

## Supplementary information

**Additional file 1: Figure S1.** DNA sequencing files of the *GNB1L* 31-bp indel. (a) Partial sequence of the *D* allele. (b) Partial sequence of the *I* allele. **Figure S2.** Electrophoresis (3.0%) patterns showing the amplification results for *GNB1L*. *DD*, *ID* and *II* are the three different genotypes, and M represents DL2000. Because the gel did not melt sufficiently, a white stain appears in the picture. **Figure S3.** Percentages of the *DD* (blue), *ID* (red), and *II* (gray) genotypes in four types. **Figure S4.** Transcription factor binding sites predicted in the *GNB1L* 31-bp indel. AliBaba 2.1 online website parameters were used such as cons = 75% and classification level K = 4. **Table S1.** Pairwise fixation index (Fst) of *GNB1L* in different chickens. Note: F2: F2 resource population (F2; *n* = 360); ND: Ningdu chickens; RW: Recessive white Rock chickens; ISA: ISA brown laying hen; GX: Guangxi chickens; WC: Wenchang chickens; QY: Qingyuan chickens; LS: Lushi chickens. **Table S2.** Association analysis of the *GNB1L* 31-bp indel with meat traits in the Xinghua × Recessive White Rock F2 populations. Note: SE = standard error of the mean; BMSF = breast muscle shear force; LMSF = leg muscle shear force; RWL = rate of water loss; CLMF = cross-sectional area of leg muscle fibers; CBMF = cross-sectional area of breast muscle fibers; BMDC = breast muscle dry matter content; BMDC = leg muscle dry matter content; BMFC = breast muscle fat content; LMFC = leg muscle fat content. **Table S3.** Details of primer pairs. Note: “–” represents a primer that is not used for genotyping.

## Data Availability

All the data and materials supporting the conclusions of the study are included in the manuscript and Additional file 1.

## Abbreviations

*GNB1L*: G protein subunit beta 1 like
indel: Insertion/deletion
SNP: Single-nucleotide polymorphism
LS: Lushi chickens
ND: Ningdu chickens
WC: Wenchang chickens
QY: Qingyuan Partridge chickens
RW: Recessive White Rock chickens
ISA: ISA Brown laying hen
GX: Guangxi chickens
F2: F2 population
XH: Xinghua chickens
PIC: Effective polymorphism information content
HWE: Hardy–Weinberg equilibrium
Ne: Allele numbers
He: Genetic indices of heterozygosity
TFs: Transcription factors
UTR: Untranslated region
SE: Standard error of the mean
BW: Body weight
SD: Shank diameter
SL: Shank length
DG: Daily gain
LWS: Live weight before slaughter
BWH: Breast width
BP: Breast depth
BSL: Body slanting length
BAW: Breast angle width
CW: Carcass weight
SFT: Subcutaneous fat thickness
FCW: Fat cingula width
EW: Eviscerated weight
LMW: Leg meat weight
WW: Wing weight
BMW: Breast meat weight
SEW: Semi-eviscerated weight
AFW: Abdominal fat weight
SIL: Small intestine length
