# Supplementary material for: A 31-bp indel in the 5′ UTR region of GNB1L is significantly associated with chicken body weight and carcass traits
Source: BMC Genet. 2020 Aug 26;21:91. doi: 10.1186/s12863-020-00900-z (PMC7450547; doi:10.1186/s12863-020-00900-z)
Supplement: Supplementary file 1 — Additional file 1: Figure S1. DNA sequencing files of the GNB1L 31-bp indel. (a) Partial sequence of the D allele. (b) Partial sequence of the I allele. Figure S2. Electrophoresis (3.0%) patterns showing the amplification results for GNB1L. DD, ID and II are the three different genotypes, and M represents DL2000. Because the gel did not melt sufficiently, a white stain appears in the picture. Figure S3. Percentages of the DD (blue), ID (red), and II (gray) genotypes in four types. Figure S4. Transcription factor binding sites predicted in the GNB1L 31-bp indel. AliBaba 2.1 online website parameters were used such as cons = 75% and classification level K = 4. Table S1. Pairwise fixation index (Fst) of GNB1L in different chickens. Note: F2: F2 resource population (F2; n = 360); ND: Ningdu chickens; RW: Recessive white Rock chickens; ISA: ISA brown laying hen; GX: Guangxi chickens; WC: Wenchang chickens; QY: Qingyuan chickens; LS: Lushi chickens. Table S2. Association analysis of the GNB1L 31-bp indel with meat traits in the Xinghua × Recessive White Rock F2 populations. Note: SE = standard error of the mean; BMSF = breast muscle shear force; LMSF = leg muscle shear force; RWL = rate of water loss; CLMF = cross-sectional area of leg muscle fibers; CBMF = cross-sectional area of breast muscle fibers; BMDC = breast muscle dry matter content; BMDC = leg muscle dry matter content; BMFC = breast muscle fat content; LMFC = leg muscle fat content. Table S3. Details of primer pairs. Note: “–” represents a primer that is not used for genotyping. [file 12863_2020_900_MOESM1_ESM.docx]

**Additional file 1**

**A 31-bp indel in the 5' UTR region of *GNB1L* is significantly associated with chicken body weight and carcass traits**

Tuanhui Ren^1,2^, Ying Yang^1^, Wujian Lin^1,2^, Wangyu Li^1,2^, Rong Fu^1,2^, Mingjian Xian^1,2^, Zihao Zhang^1,2^, Wen Luo^1,2^, Xiquan Zhang^1,2*^


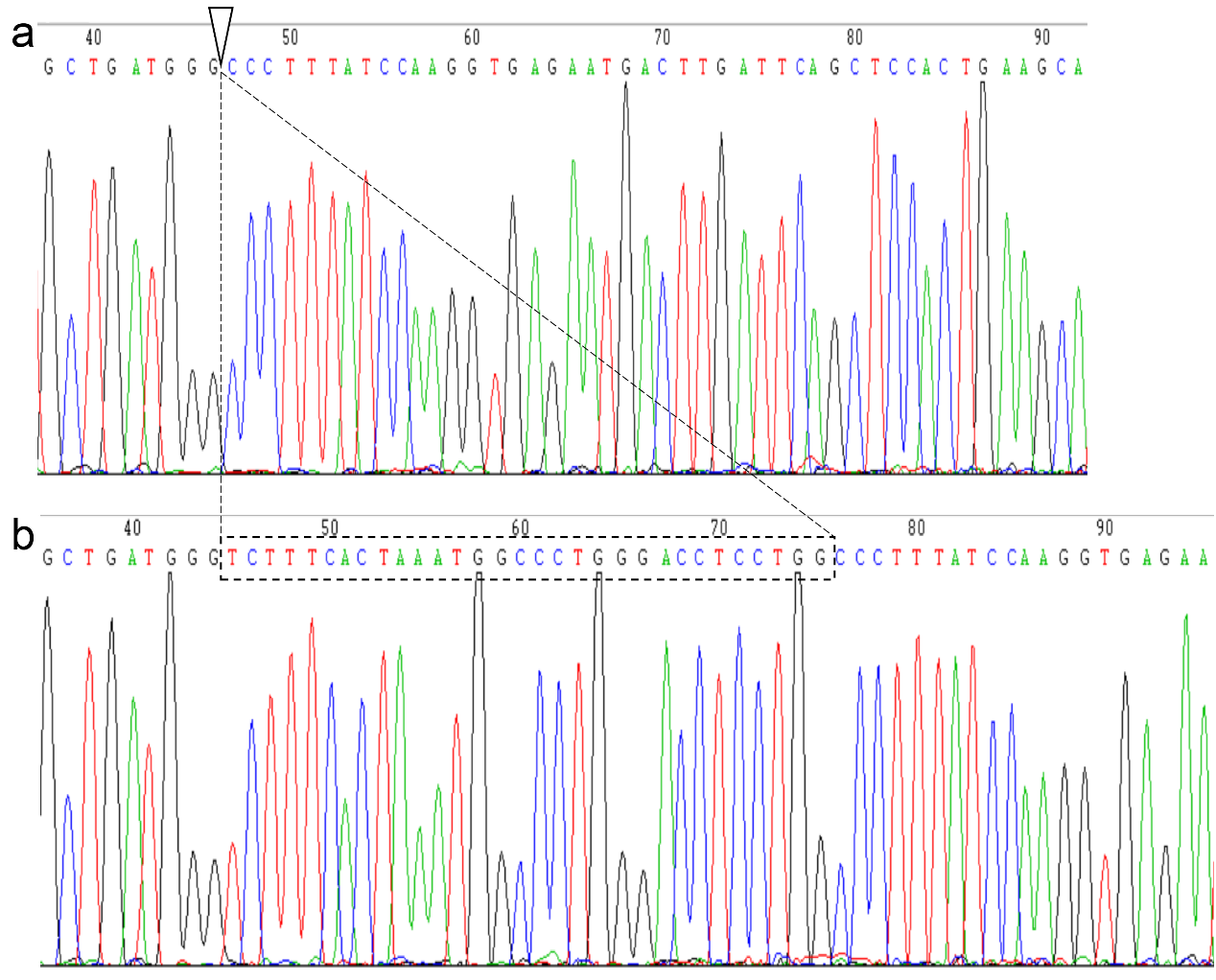


**Figure S1.** DNA sequencing files of the *GNB1L* 31-bp indel. (a) Partial sequence of the *D* allele. (b) Partial sequence of the *I* allele.


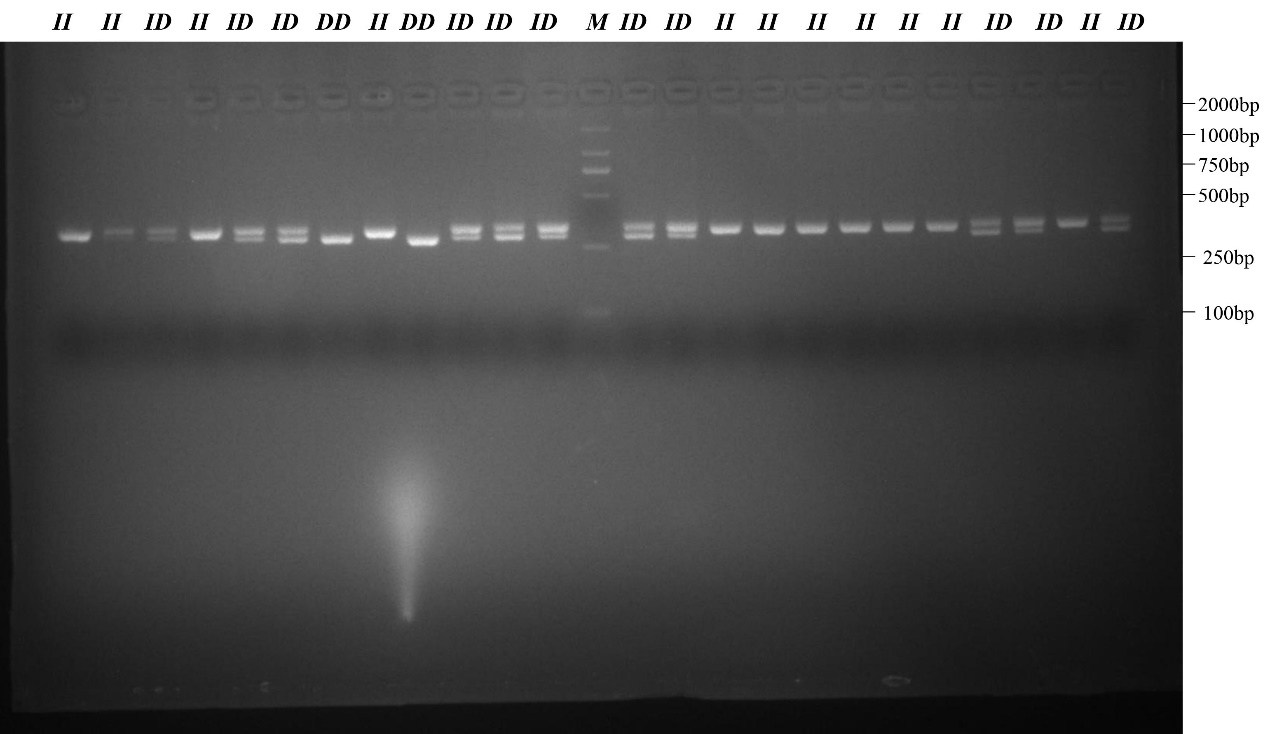


**Figure S2.** Electrophoresis (3.0%) patterns showing the amplification results for *GNB1L*. *DD*, *ID* and *II* are the three different genotypes, and M represents DL2000. Because the gel did not melt sufficiently, a white stain appears in the picture.


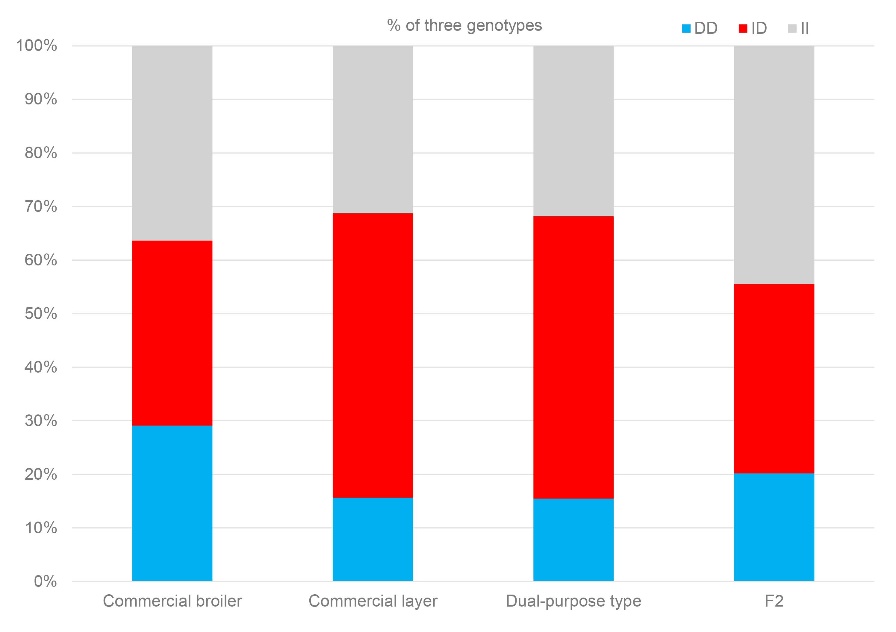


**Figure S3.** Percentages of the *DD* (blue), *ID* (red), and *II* (gray) genotypes in four types.


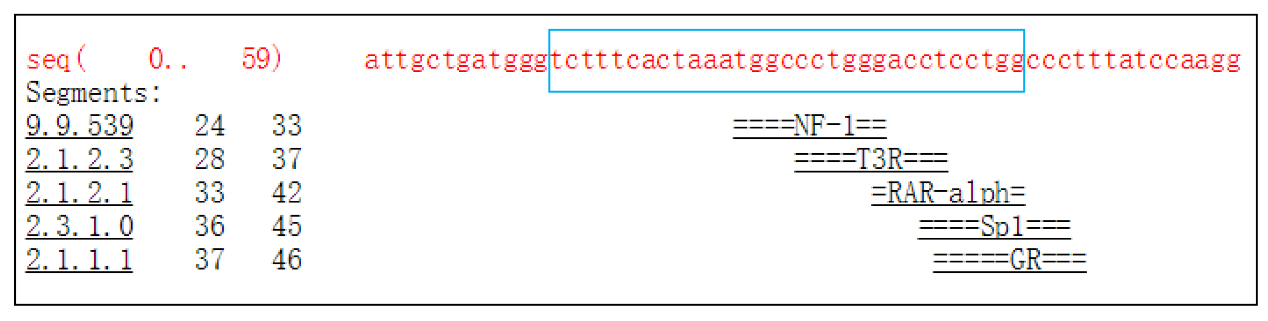


**Figure S4.** Transcription factor binding sites predicted in the *GNB1L* 31-bp indel. AliBaba 2.1 online website parameters were used such as cons = 75% and classification level K = 4.

**Table S1.** Pairwise fixation index (Fst) of *GNB1L* in different chickens.

|  | F2 | WC | LS | RW | QY | ND | ISA |
| --- | --- | --- | --- | --- | --- | --- | --- |
| WC | 0.003 |  |  |  |  |  |  |
| LS | 0.023 | 0.027 |  |  |  |  |  |
| RW | 0.004 | 0.000 | 0.022 |  |  |  |  |
| QY | 0.000 | 0.005 | 0.055 | 0.008 |  |  |  |
| ND | 0.000 | 0.003 | 0.042 | 0.005 | 0.000 |  |  |
| ISA | 0.001 | 0.001 | 0.035 | 0.002 | 0.002 | 0.001 |  |
| GX | 0.000 | 0.008 | 0.061 | 0.011 | 0.000 | 0.001 | 0.004 |

Note: F2: F2 resource population (F2; n = 360); ND: Ningdu chickens; RW: Recessive white Rock chickens; ISA: ISA brown laying hen; GX: Guangxi chickens; WC: Wenchang chickens; QY: Qingyuan chickens; LS: Lushi chickens.

**Table S2.** Association analysis of the *GNB1L* 31-bp indel with meat traits in the Xinghua × Recessive White Rock F2 populations.

| Traits | Mean±SE | | | *P*-value |
| --- | --- | --- | --- | --- |
|  | *DD* | *ID* | *II* |  |
| LMSF | 78.4±3.3 | 74.6±2.5 | 72.6±2.2 | 0.338 |
| BMSF | 33.7±1.2 | 32.4±0.9 | 33.3±0.8 | 0.648 |
| RWL (%) | 17.9±1.7 | 20.34±1.8 | 18.6±1.4 | 0.592 |
| CLMF | 19635.4±516.5 | 19845.5±367.7 | 19680.7±376.1 | 0.93 |
| CBMF | 22018.7±579.2 | 21320.0±412.3 | 21831.4±421.7 | 0.547 |
| BMDC (%) | 26.0±0.2 | 26.1±0.2 | 26.2±0.2 | 0.681 |
| BMDC (%) | 25.0±0.3ab | 24.9±0.3a | 25.3±0.3b | 0.046 |
| BMFC (%) | 0.9±0.1 | 0.9±0.1 | 0.9±0.1 | 0.764 |
| LMFC (%) | 3.6±0.3 | 3.3±0.3 | 3.8±0.3 | 0.05 |

Note: SE = standard error of the mean; BMSF = breast muscle shear force; LMSF = leg muscle shear force; RWL = rate of water loss; CLMF = cross-sectional area of leg muscle fibers; CBMF = cross-sectional area of breast muscle fibers; BMDC = breast muscle dry matter content; BMDC = leg muscle dry matter content; BMFC = breast muscle fat content; LMFC = leg muscle fat content.

**Table S3.** Details of primer pairs.

| Primers (5'-3') | Sizes (bp) | Tm (℃) | Genotype (bp) |
| --- | --- | --- | --- |
| GNB1L-F: TGCTGCACTGGAGCTAACAA | 332 | 61 | *DD:* 332, *ID:* 332+301, *II:* 301 |
| GNB1L-R: GCTCAGCCAGTCAGTGGTAG |  |  |  |
| GNB1L-DF: GGATCTGCCTGTGGGATTTA | 158 | 60 | - |
| GNB1L-DR: CAAAACCTGAACCTCCTCCA |  |  |  |
| actin-F: GACTGACCGCGTTACTCCCA | 166 | 60 | - |
| actin-R: CCAACCATCACACCCTGATGTC |  |  |  |

Note: “–” represents a primer that is not used for genotyping.
